# Is Genetic Risk for Sleep Apnea Causally Linked With Glaucoma Susceptibility?

**DOI:** 10.1167/iovs.63.1.25

**Published:** 2022-01-20

**Authors:** Nathan Ingold, Adrian I. Campos, Xikun Han, Jue-Sheng Ong, Puya Gharahkhani, David A. Mackey, Miguel E. Rentería, Matthew H. Law, Stuart MacGregor

**Affiliations:** 1 Department of Genetics & Computational Biology, QIMR Berghofer Medical Research Institute, Brisbane, Queensland, Australia; 2 School of Biomedical Sciences, Faculty of Health, Queensland University of Technology, Brisbane, Queensland, Australia; 3 School of Biomedical Sciences, Faculty of Medicine, The University of Queensland, Brisbane, Queensland, Australia; 4 Centre for Ophthalmology and Visual Science, Lions Eye Institute, University of Western Australia, Nedlands, Western Australia, Australia

**Keywords:** glaucoma, sleep apnea, Mendelian randomization, genetics

## Abstract

**Purpose:**

Observational studies have suggested that individuals with pre-existing sleep apnea (SA) have up to double the risk of developing glaucoma than individuals without SA. Understanding risk factors for glaucoma is important to assist with well-structured screening, early intervention, and efficient allocation of specialist consultation. The objective of this study is therefore to use genetic data to determine whether SA is a causal risk factor for glaucoma.

**Methods:**

Two-sample Mendelian randomization (MR) analyses were performed to assess the association between genetically predicted SA and glaucoma susceptibility using genome-wide association study (GWAS) of 25,062 SA cases, 313,372 controls derived from 23andMe and summary data from a glaucoma GWAS meta-analysis (20,582 cases, 119,318 controls), including individuals of European descent, mainly from the UK Biobank.

**Results:**

Inverse variance weighted regression of genetic susceptibility for SA on risk of glaucoma revealed no strong evidence for an association between SA and glaucoma (OR = 0.95, 95% confidence intervals = 0.84–1.07), results were consistent across all MR predictors.

**Conclusions:**

We found little genetic evidence supporting a causal association between SA and glaucoma. Our results refute the possibility of a large effect (glaucoma OR > 1.5 per doubling of odds on SA) between SA and glaucoma.

Sleep apnea (SA) is the frequent obstruction of the upper airways during sleep. Within SA, obstructive SA is by far the most prevalent—estimated to affect 20% to 30% of adults.^1^ Intermittent SA is also believed to be linked with increased blood pressure, and decreased blood flow and hypoxia of the optic nerve that could lead to optic nerve neuropathy.^2^^,^^3^ Damage to the optic nerve is a hallmark of glaucoma, a leading cause of blindness worldwide.^4^ SA is, therefore, commonly speculated to be a risk factor for glaucoma.^5^^,^^6^

The most prevalent form of glaucoma estimated 76.0 million individuals in 2020 affected by the disease.^7^ In most cases, glaucoma arises as a result of elevated intraocular pressure (IOP), which leads to irreversible damage to the optic nerve head.^8^^,^^9^ Early intervention is the most effective treatment of glaucoma and prevents subsequent blindness.^10^ Thus, identifying causal risk factors for glaucoma may be valuable for early detection and prevention.

To date, findings on the relationship between SA and glaucoma have been inconclusive. A recent meta-analysis of the association between glaucoma and SA pooled six case-control and nine cross-sectional studies and found that individuals diagnosed with SA on average had a two-fold increase in the risk of developing glaucoma with odds ratios (OR) of 1.96 (95% confidence interval [CI] = 1.37–2.80) and OR = 1.41 (95% CI = 1.11–1.79), respectively.^6^ However, findings from other studies concluded no clear relationship between the two diseases.^11^^,^^12^ Results from a cohort study showing a moderate association between SA and glaucoma (OR = 1.67, 95% CI = 1.30–2.17^13^) are conceivably less biased by reverse causality. Yet, other forms of ascertainment and selection bias cannot be excluded entirely.

Genetic-based instrumental variable (IV) techniques such as Mendelian randomization (MR) are promising alternatives to assess potential causal relationships between traits. They are less susceptible to bias generated from confounding, selection bias and reverse causality that commonly hamper traditional epidemiological studies.^14^ As genetic variants are randomly assorted at meiosis, MR studies are by design randomized, blinded and unbiased, akin to randomized control trials.^15^ To perform MR, suitable genetic IVs (generally single nucleotide polymorphisms [SNPs]) must first be identified through genome-wide association studies (GWAS), which to date have been underpowered to detect any genetic loci associated with SA, a highly under-diagnosed trait.^16^^,^^17^ Following recent advances in GWAS analysis methods, Campos et al.^18^ conducted multi-trait analysis of GWAS (MTAG), combining data on SA and related traits. MTAG boosts discovery power of GWAS of the trait of interest by leveraging power from genetically similar traits. This MTAG analysis identified 39 genetic loci robustly associated with SA, with the loci replicated in a large independent SA case-control study.^18^ These discoveries enable reappraisal of the possibility of revisiting the controversial relationship between SA and glaucoma through an MR framework. In this study, we attempt to clarify whether there is genetic evidence supportive of a causal relationship between SA susceptibility and the risk of developing glaucoma using a two-sample MR framework and compare these findings against previous observational findings.

## Methods

### Study Overview

Using instruments curated from the most recent sleep apnea GWAS^18^ and data from the most recent glaucoma GWAS, we conducted a two-sample MR analysis to evaluate whether genetically predicted higher susceptibility towards SA increases the risk of glaucoma.^19^

### Data Source: Sleep Apnea Summary Statistics

The SA multi-trait discovery GWAS used a total number of 25,062 SA cases, 172,050 snoring cases and 313,372 controls (N_Total_ = 510,484) across a variety of cohorts. All cohorts used were filtered to include only European descent individuals using principal component analysis, with principal components fitted as covariates to account for any residual stratification. SA data were collected through International Classification of Diseases Tenth Revision (ICD-10; N = 14,952 [59.9% of the cases]) codes, accessing primary care records (N = 4726 [18.9%]) or through self-reporting via questionnaire (N = 5325 [21.2%]). The three cohorts that included self-report questionnaire data for SA had a mixture of participants answering “sleep apnea” when asked about illnesses they have been told they have by a doctor (UK Biobank [UKBB]), a question relating to stopping breathing during sleep (Canadian Longitudinal Study of Aging) and a question on how many nights per week have they been told that they struggled for breath (Australian Genetics of Depression Study). All snoring data was collected through self-report questionnaire with similar questions (e.g., “how many nights or days per week have you had or been told you had loud snoring?).”^18^

To avoid any biases due to sample overlap (described later), in this analysis we specifically used the genetic effect size estimates (i.e., beta coefficient and its respective standard error) derived from an independent 23andMe case-control cohort of SA, which was used for replication by Campos et al.^18^ The 23andMe cohort comprised 175,522 self-report SA cases and 1,301,803 controls (see Campos et al.^18^ for each of the 39 SA-associated variants).

### Data Source: Glaucoma Summary Statistics

The summary statistics for glaucoma in Europeans were provided by Craig et al.,^19^ who used the multi-trait approach (MTAG) to analyze glaucoma, IOP and vertical cup-disc ratio (VCDR) data from the UKBB and the International Glaucoma Genetics Consortium. The GWAS findings were derived from 7947 glaucoma cases, 119,318 glaucoma-free controls, 133,492 IOP measurements, and 90,939 VCDR measurements. The multi-trait approach produces log Odds Ratio (log(OR)) estimates that are specific to glaucoma but with smaller standard errors—akin to leveraging genetically correlated phenotypes to perform a standard glaucoma GWAS the equivalent of 20,582 glaucoma cases and 119,318 controls.^19^ Technical details for the MTAG have been described elsewhere.^20^ Glaucoma participants were defined by ICD-10 codes and self-reporting through questionnaires.

### Selection of Instrumental Variable for SA

The IVs comprised 39 independent SNPs associated with SA (*P* < 5e-8; linkage disequilibrium R^2^ = 0.05); all replicated in the independent 23andMe validation cohort as reported in Campos et al.^18^ To minimize potential winner's curse bias in the MR estimates due to sample overlap^21^ of UKBB individuals who appear in both the SA and glaucoma study, we used the effect sizes and standard errors from the 23andMe SA analysis (there was no sample overlap between the 23andMe SA GWAS and the glaucoma GWAS).

### Estimation of Phenotypic Variance Explained by Instruments and Weak Instrument Bias

Using weak IVs can violate the MR core strong instrument assumption and induce weak instrument bias.^22^ To assess instrument strength, an F statistic for each IV was calculated. A combined F-statistic > 10 means the IVs are considered to be robust MR instruments.^22^ To calculate the F-statistic we first determined the phenotypic variance captured by each IV. The following equation was used to estimate the proportion of phenotypic variance explained by IVs on the observed scale.^23^

$$\;R^{2}=\frac{2\beta^{2}\;MAF\left(1-MAF\right)}{2\beta^{2}\;MAF\left(1-MAF\right)\;+\;s{e_{\beta }}^{2}\;2n\;MAF\left(1-MAF\right)}$$

Where *MAF* is the minor allele frequency of the IV, β is the beta coefficient effect size estimate, se is the standard error of β, and *n* is the sample size.

To assess the IVs as strong MR instruments, the combined instrument F statistic was calculated using the following equation:

$$F=\;\frac{R^{2}\left(n\;-\;1\;-k\right)}{\left(1\;-\;R^{2}\right)k}$$

where *R*^2^ is the phenotypic variance explained, *n* is the sample size and *k* is the number of IVs.^24^^,^^25^

The main determinants of power in an MR study are the variance captured by the IVs and the sample size of the outcome GWAS (glaucoma GWAS). A well-powered study will provide sufficiently small confidence intervals that one can assess what effect sizes are plausible given the data.

### Statistical Analysis

After evaluating IV strength and before the MR analysis, we assessed the validity of our SNP instruments against key MR model assumptions (Supplementary Methods). We estimated our MR association using the generalized summary-data-based Mendelian randomization (GSMR) framework—a tool from Genome-wide Complex Trait Analysis (GCTA),^26^ which additionally models the precision in the exposure beta estimates and adjusts for heterogeneous SNP-outliers through HEIDI-filtering (Supplementary Methods). We also then applied the multiplicative random effects model inverse variance weighted (IVW) model to combine individual Wald estimates into a combined association estimate. To evaluate potential bias in the IVW results due to weak instrument bias (which violates a key MR assumption; see Supplementary Methods for MR assumptions), we applied several alternative MR models (namely MR-Egger, MR-weighted median, simple mode, and weighted mode) to strengthen evidence for MR causality. Technical details on these methods have been previously described.^14^^,^^27^^,^^28^ In addition, for ease of interpretation given a binary exposure, all presented ORs and β coefficients are scaled so that the OR estimates reflect the effect size on glaucoma risk per doubling of odds on SA; this was done by multiplying the IVW estimate (in log[OR]) by log(2)∼ = 0.693^29^^,^^30^ to reflect a scaled β coefficient then taking the exponential for a scaled OR.

A Z test was also performed between the IVW from the MR analysis and the effects derived from previously published observational results. For this β coefficients and standard errors (SE) were derived from OR values provided from previously reported observational results, differential Z were derived by the following:

$$Z\;=\frac{\beta_{IVW}\;-\;\beta_{Obs}}{\sqrt{se_{IVW}^{\;2}}\;+\;se_{Obs}^{\;2}\;}$$

where β_*IVW*_ is the effect estimate of the IVW, β_*Obs*_ is the effect estimate of the observational test, $se_{IVW}^{\;2}$ is the SE of IVW squared, $se_{Obs}^{\;2}$ is SE of the observational effect estimate squared.

### Software

R-3.6.2^31^ was used for all statistical analyses and illustrations. Specifically, two R packages “TwoSampleMR” and “MRInstruments,” both curated from the MR-Base platform (https://www.mrbase.org/), were used to perform the MR analyses along with sensitivity analyses and for generating MR forest/scatter plots.^28^ The GSMR-GCTA^26^ analysis was performed using the GCTA software^32^ within a UNIX/BASH environment. The results of GSMR and the forest plot of OR values were plotted using native R-3.6.2 plot function.

## Results

### Mendelian Randomization

We took 39 SNPs exceeding genome-wide significance in the discovery GWAS meta-analysis,^18^ and which replicated in the independent 23andMe data set, ensuring that collectively all SNPs constitute a strong instrument for MR (first assumption of MR). All GWAS included in the Campos et al.^18^ meta-analysis were filtered to only include individuals of European descent, and controlled for age and sex (second assumption of MR; for more details of GWAS samples and filtering see reference 18). By using HEIDI-outlier test statistics to detect variants with high heterogeneous effect sizes, we excluded five SNPs as heterogeneous outliers (third assumption of MR). The remaining 34 SNPs went on to constitute the IVs in the MR analysis. The proportion of variance in SA on the observed scale explained by the 34 SNP instruments (R^2^; calculated with 23andMe data) was estimated to be 1.02%, (this is ample for strong MR analysis, given the large sample size of the SA GWAS used).

**Figure 1.**
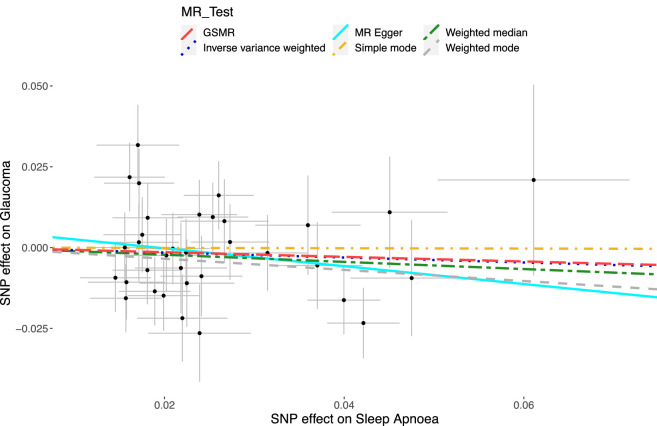
Scatter plot of each SNP's respective effect size for sleep apnea and glaucoma. The X-axis refers to the estimated magnitude of association (log(OR)) of each of the 34 IV single nucleotide polymorphisms (SNPs) on sleep apnea, whereas the Y axis refers to the magnitude of association (log(OR)) of each IV on glaucoma risk. The SEs are plotted for each point. The regression lines represent (1) inverse variance weighted (*blue dots*; IVW), which is the primary regression with no adjustment for pleiotropic effect; (2) MR Egger (*turquoise* *full line*; Mendelian randomization Egger), which accounts for directional pleiotropy; (3) weighted median (*green long-dash dot*), which provides robust point estimates even when up to 50% of the IVs are invalid instruments; (4) simple mode (*yellow short-dash dot*), providing the effect estimate based on the mode of the Wald-type estimates; (5) weighted mode (*gray short-dash*), assigns SE-based weightings to each SNP of the simple mode method; and (6) GSMR (*red long-dash*), which is similar to IVW after removing the HEIDI outliers. Note: Because of the similar effect estimates between IVW and GSMR, the lines overlap and maybe misconstrued as one “dot-dash” line; they are in fact two separate lines.

**Figure 2.**
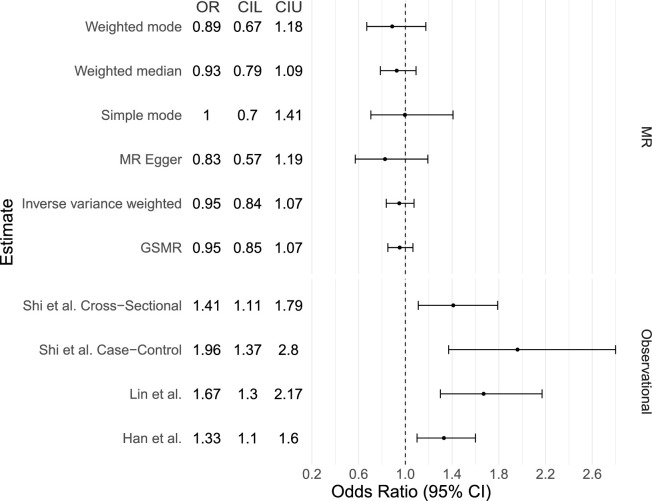
Forest Plot of the estimated odds ratios from our Mendelian randomization analysis and from previously reported observational studies. Forest plot presenting OR (representing a doubling of odds of SA on glaucoma) and lower (L) and Upper (U) 95% CI estimates for both the MR results (per doubling of odds) and observational findings (from logistic regression; Shi et al.^6^) and a hazard ratio (HR) estimate for glaucoma from Han et al.,^33^ based on time-to-event analysis using UKBB data and a population-based matched-cohort study.^13^

Regression of the SA and glaucoma β coefficients using IVW resulted in β = −0.07, SE = 0.09, *P* value = 0.40 (Supplementary Table S4; Fig. 1). Deriving an OR to reflect a doubling in genetic odds of SA resulted in OR = 0.95, 95% confidence intervals (CIs) = 0.84–1.07 (Supplementary Table S4, Fig. 2), which represents no causal association between the two traits. Using MR-Egger to account for potential unbalanced horizontal pleiotropic effects of the IVs did not meaningfully change the results (OR = 0.82, 95% CI = 0.57–1.19, *P* value = 0.32). Furthermore, the MR-Egger intercept, which if greater than 0 can indicate the presence of horizontal pleiotropy, was 0.005 with a *P* value of 0.43, indicating no pleiotropic effect of the IVs. The weighted median MR estimate, which remains valid in the presence of a large proportion of invalid instruments (i.e., up to 50%), yielded similar results to the IVW analysis (OR = 0.93, 95% CI = 0.79–1.09, *P* value = 0.36). Using the same 34 IVs in GSMR gave similar results with an OR of 0.95 (95% CI = 0.85–1.06, *P* value = 0.38; Supplementary Table S4; Fig. 1; Fig. 2).

Comparing the result from IVW from the MR analysis to the previously published observational ORs, a Z-score differentiation test (Methods) revealed our IVW estimate was significantly smaller than all four studies included in Figure 2. Specifically, IVW comparisons with Shi et al.^6^ (case-control) revealed a significant decrease (*P* = 1.65E-4), as did Shi et al. (cross-sectional) (*P* = 3.86E-3), Han et al.^33^ (*P* = 2.93E-3), and Lin et al.^13^ (*P* = 1.3E-4).

## Discussion

This study is the first to use MR to evaluate the genetic causality between SA and glaucoma. We found no evidence supportive of a causal relationship between SA and glaucoma, which is contrary to many previous observational reports.^6^^,^^13^ Furthermore, our results are consistent across estimates from pleiotropy/heterogeneity-robust MR techniques, suggesting that previously observed large positive findings between SA and glaucoma are likely misrepresenting the true relationship.

While the meta-analysis combines sleep apnea with snoring data, we are confident that the IVs are not just recapitulating the effect of snoring on glaucoma as Supplementary Plot S1 shows the 34 IVs have a strong correlation in effect size (r^2^ = 0.88) between snoring and sleep apnea in the UKBB. The 34 IVs satisfy all three assumptions of MR (see Supplementary Methods), this is important as it provides confidence that the results are truly measuring the effect of SA on glaucoma and not an unmeasured/unknown artifact that is driving the results.

Both cohorts used in this study were very large, which enabled well-powered analysis, with 25,062 SA cases and 313,372 controls, combined with a GWAS of glaucoma with an effective sample size of 20,582 cases and 119,318 controls.^19^ Furthermore, the IVs selected in this study were replicated in an independent dataset,^18^ reducing the chance of potential bias from winner's curse. In MR studies, the primary determinants of power are the variance explained by the SNP instruments (1.02% here) and the sample size in the outcome GWAS (glaucoma here); in combination our variance explained and the sample size resulted in good precision (narrow confidence intervals) for our MR estimate. We can therefore say with confidence that our analysis has ample power to detect moderate or large effects of SA on glaucoma. Small effects of SA on glaucoma remain possible because these cannot be ruled out by the confidence intervals we obtained.

The estimated effect size between genetically predicted liability on SA and glaucoma is very small (OR = 0.95 95% CI = 0.83–1.07), providing no support for a causal association, with similar findings derived from alternative pleiotropy-robust models. The doubling of odds OR estimated in MR is reflecting a large change in the liability of SA, our point (OR) estimates were smaller than those derived from traditional logistic regression models used in observational studies, with no overlap in the CIs (Fig. 2).^6^^,^^13^ Because findings from MR analyses are less likely to be influenced by the effect of confounding or reverse causality, our results suggest that previous observational associations may be influenced by unmeasured confounding factors. An example of a potential confounder may be the systematic selection of more instances of a trait such as diabetes in the case group than the control group; diabetes is associated with both SA and glaucoma.^34^^,^^35^

Although we can conclude that higher genetic liability for SA does not translate to a large effect on glaucoma risk, our findings do not rule out the possibility of a very small causal effect of SA on glaucoma. To further illustrate this point, recently Han et al.^33^ conducted an observational time-to-event analysis over 8 years using UKBB data (N = 502,505), and reported a moderate association between SA and glaucoma (hazard ratio for glaucoma = 1.33, 95% CI = 1.10–1.60); Our null MR estimate is significantly different from the estimate of Han et al.^33^ (*P* = 2.93E-3). Our MR estimates could indicate that if a causal effect exists, this could be slightly protective. However, a more probable explanation if a causal effect exists at an end point that this study is underpowered to detect is that the increase in glaucoma risk per SD change in risk of SA is very small (between OR 1.0–1.07). Under either circumstance, this should not alter management of either condition. We believe the scaling of OR to a doubling of odds (as described in methods) allows for the most accurate comparison of our results to previously published results, given the null result, the specific scaling used does not change our conclusions.

Apart from issues on confounding, another possible limitation on observational studies reporting very large associations could be due to selection bias,^36^ where the selection of individuals with SA within the study introduces systematic difference between study population and regular population, thereby biasing the results. We speculate that the selection bias could be explained by an age effect, where SA and glaucoma are more likely to co-occur in older individuals. Because variants for SA are assigned at meiosis, this is unlikely a major limitation for MR inference.

Notably, a limitation to this study is the lack of good power to detect small associations (i.e., OR∼1.1); the IVs in our study captured an estimated 1.02% of the total phenotypic variance on SA explained by the 34 SNPs. As revealed by our 95% CI for the MR estimates, we are unable to reliably rule out a potential causal effect of OR∼1.1, keeping in mind that this is for a large change in the liability of SA (i.e., doubling of odds for SA).

Also, the correction of the effect of body mass index (BMI) on SA but not on glaucoma, which could lead to a type II error in our analysis. In practice, this is unlikely a major concern because there is no established evidence for BMI being a major risk factor for glaucoma^37^; hence, adjusting for BMI in one GWAS and not the other will not systematically bias the effect. Although the adjustment for BMI in the SA GWAS can conceptually remove confounding signals between SA-associated variants and obesity (which is the strongest risk factor for SA), we acknowledge that there is potential for collider bias in the resultant MR estimate.^38^ To examine this possible bias, we calculated correlation between the 34 SA SNP effect estimate between those reported in Campos et al. (i.e., not adjusted for BMI) and those used in the present analysis to avoid sample overlap of UKBB participants who appear in both samples (from 23andMe replication; adjusted for BMI). The effect estimates showed very strong correlations (*r*^2^ = 0.85), suggesting minimal influence on our MR inference.

Another limitation is the use of self-reported SA data in this study. Self-reported data are less reliable than data collected through other means (i.e. by using ICD-10 code) and increase the chance that the instruments used in our analysis are not truly associated with SA. Replication of the GWAS results of Campos et al.^18^ in 23andMe reduces the chance of false-positive results, and gives us confidence that the MR instruments are legitimate. This limitation has been discussed further by Campos et al.^18^ The effect size estimates in this study were taken from 23andMe that were derived from self-reported data, which could potentially systematically bias all beta coefficients toward the null, and result in a false-negative result. However, the high *r*^2^ (0.85) between the 23andMe and original meta-analysis estimates (the latter being derived largely from ICD-10 and GP records) suggests that this is not the case.

Although we use samples that have been filtered to include only European ethnicity, it is important to note that ethnicity is an important factor in glaucoma. This is therefore a limitation of this study, and additional work should be carried in other ethnicities.

Another limitation to consider is survival bias induced when incorporating age-related traits (glaucoma) in MR.^39^ Survivorship bias is brought about by missing individuals who would have gotten glaucoma but died of something else first; this could bias the result toward the null. This highlights an avenue for future work using longitudinal cohorts to determine whether genetically predicted SA affects survival.

Finally, we cannot infer conclusively whether SA contributes to optic neuropathy via specific mechanisms (e.g., thinning of the retina) as the study only evaluated overall risk of glaucoma based on genetic data. However, given the strong genetic correlation between glaucoma and VCDR,^19^ it is unlikely that a genetic predisposition toward risk of SA contributes to the causal mechanisms linking to optic neuropathy. However, future studies with genetic data dissecting specific biological mechanisms for predisposition on SA would be warranted to revisit this relationship. Further MR work with SA and specific subtypes of glaucoma will also be warranted.

## Conclusion

We found little genetic evidence supporting a causal association between SA and glaucoma. Although genetically derived estimates are conceivably less precise, our results are not consistent with the estimates obtained from previous observational studies. Hence, for a relatively large change in risk of developing SA, our findings are able to confidently exclude all but a very small potential increase in risk of glaucoma, which is double negative likely not of clinical relevance.

## Supplementary Material

Supplement 1
