# Supplementary material for: Is Genetic Risk for Sleep Apnea Causally Linked With Glaucoma Susceptibility?
Source: Invest Ophthalmol Vis Sci. 2022 Jan 20;63(1):25. doi: 10.1167/iovs.63.1.25 (PMC8787584; doi:10.1167/iovs.63.1.25)
Supplement: Supplement 1 [file iovs-63-1-25_s001.pdf]

Supplementary appendix

Is genetic risk for sleep apnoea causally linked with glaucoma susceptibility?

Authors:

Nathan Ingold*[1,2], Adrian I. Campos[1,3], Xikun Han[1,3], Jue-Sheng Ong[1], Puya Gharahkhani[1], David A. Mackey[4], Miguel E. Rentería[1,2,3], Matthew H. Law[1,2], Stuart MacGregor[1]

Contents

- Supplementary methods
- Evaluating the validity of Mendelian randomisation assumptions
- Elaboration of sensitivity analyses (MR models)
- Supplementary Table 1. Phenotype definition for SA (across the 5 studies)
- Supplementary Table 2. Phenotype definition for Glaucoma
- Supplementary Table 3. Mendelian randomisation estimates of the association between sleep apnoea and glaucoma risk
- Supplementary Table 4. Single nucleotide polymorphisms associated with sleep apnoea used in this study
- Supplementary References

Supplementary methods

Generalised Summary-data-based Mendelian Randomisation (GSMR)

The following bash syntax was used to generate the GSMR estimates, using GCTA version 1.91.7beta (8 Oct 2018)[1]:

```
gcta64 \
 --bfile ${ref_file} \
  --gsmr-file ${exposureGWAS} ${outcomeGWAS} \
  --gsmr-direction 0 \
  --gwas-thresh 0.05 \
  --threads 3 \
  --effect-plot \
  --out
```

The bfile directs to an in-house UK Biobank linkage disequilibrium reference panel 4990 randomly selected individuals. gsmr-file contains the paths to the files containing the genetic data for sleep apnoea and glaucoma, formatted for GSMR (see for more details on file formatting: https://cnsgenomics.com/software/gcta/#Mendelianrandomisation). gsmr-direction is set to 0, indicating that MR analysis is run once with sleep apnoea as the exposure. gwas-thresh is the defining the P value for selection of independent SNPs for clumping. As the results were derived from the independent SNPs tested for replication in 23andMe, the replication P threshold of 0.05 was used. Finally the GSMR HEIDI outlier P value threshold is not set, so it is therefore the default of 0.01.

MR-Base

The following script was used to generate, weighted median, weighted mode, simple mode, inverse variance weighted and Mendelian Randomisation Egger estimates, using TwoSampleMR_0.5.4 and MRInstruments_0.3.2 [2], R version 3.6.2 was used for all analysis:

```
#harmonise the exposure and outcome
 dat <- harmonise_data(
  exposure_dat = expo,
  outcome_dat = out, action = 2)

  ##perform MR
  res_dat <- mr(dat)
```

'expo' and 'out' are both files containing the relevant SNP, beta, standard error and allele data for sleep apnoea and glaucoma respectively. 'action = 2', tries to infer positive strand alleles, using allele frequencies for palindromes. See https://github.com/MRCIEU/TwoSampleMR for more details.

Evaluating the validity of Mendelian randomisation assumptions

In order to perform valid MR analysis, the genetic instruments must satisfy three important assumptions [3]:

1. The genetic instruments are associated with the exposure.
2. The genetic instruments used are independent of any confounding variables that influence both exposure and outcome.
3. The genetic instruments used only affect the outcome through the exposure.

The first assumption is satisfied by selecting SNPs that reach or exceed genome-wide significance (P value $< 5 \times 10^{-8}$) in the SA meta-analysis, and by excluding weak instrument bias (discussed later in methods).

Assumptions two and three both refer to forms of confounding, and each are accounted for in different ways. Assumption two can be violated by confounding via unconsidered variables such as population stratification. Bias due to ancestry can be satisfied by controlling for population structure, which has been done in both the SA and glaucoma GWAS used in this analysis.

The third assumption is potentially violated in this analysis due to the pleiotropic nature of SNPs. To address this, we measured and controlled for pleiotropy in multiple ways (described below).

Elaboration of sensitivity analyses (MR models)

The HEIDI-outlier method was applied to detect potentially pleiotropic SNPs where the estimates of beta coefficient were significantly different from expected effect for a causal model, and remove them from the analysis[1]. Q-statistics were calculated for the genetic instruments to identify heterogeneity, which can be an indicator of pleiotropic effect; any IV contributing to significant levels of heterogeneity was excluded. Finally, the IVW regression was adjusted by multiple methods to account for potential pleiotropic effects. MR-Egger was used as a sensitivity analysis by adjusting the IVW method causal effect estimate so that the intercept is no longer 0, but instead reflects the estimated pleiotropic effect of the IVs used; an MR-Egger intercept deviating from 0 can indicate pleiotropy[4]. Weighted median analysis was also performed which provides an estimate of effect when assuming ~50% of the IVs weights are invalid. The simple mode clumps SNPs into groups of similar effects, and returns the effect estimate of the cluster with the largest number of SNPs, the weighted mode is an extension of the simple mode, but weights each SNP by its outcome effects MR methods are further discussed here (Hemani et al. 2018)

Supplementary Tables

Supplementary Table 1. Phenotype definition for SA (across the 5 studies)

Cohort	Sleep Apnoea (Cases/Controls)	Snoring (Cases/Controls)	Sleep apnoea Phenotype definition
UK-Biobank	7902/248 112	152 303/256 014	General practitioner records, ICD10, SR (reported in a verbal interview)
CLSA	3391/14 966	6852/11 575	SR (“stop breathing in sleep”)
AGDS	1517/8842	4450/5909	SR (“During the last month, on how many nights or days per week have you had or been told that your breathing stops or you choke or struggle for breath?”)
FinnGen	9096/57 120	4270/61 946	ICD10,ICD9
Partners Biobank	3102/16 945	4175/15 872	Electronic Health Records

Supplementary table 1: show the numbers of cases and controls of sleep apnoea and snoring for each cohort in the Campos et al. meta-analysis[5], and how each cohort defines sleep apnoea (for all cohorts snoring was defined through self-report). AGDS is the Australian genetics of depression study, CLSA is the Canadian longitudinal study of aging, SR is self-report (with the specific question in ()). For more details see Campos et al (https://www.medrxiv.org/content/10.1101/2020.09.29.20199893v1) [5].

Supplementary table 2: -Phenotype definition for Glaucoma

Phenotype	(Cases/Controls) / N	Phenotype definition
Glaucoma	(7947/119318)	ICD10, SR
IOP	133 492	Corneal compensation IOP measurement
VCDR	90 939	Non-stereo fundus images supplied by ophthalmologists

Supplementary table 2: shows the number of cases and controls for glaucoma and the total number of individuals with intraocular pressure measurement (IOP) and the total number of individuals with vertical cup to disc ratio measurements (VCDR) that were included in the glaucoma meta-analysis[6]*. We also report how each phenotype was defined; SR is self-report.*

Supplementary Table 3. Single nucleotide polymorphisms associated with sleep apnoea used in this study

SNP	A1	A2	Beta	SE	G Beta	G SE	Linked traits	Source, PMID	Nearest gene
rs10075809	C	A	0.0158	0.004282	-0.0107	0.01154	Basal metabolic rate, Weight, Height,	Neale B,	*CEP120*
rs11075985	C	A	-0.0172	0.003907	-0.0199	0.01061	Body mass index, Type II diabetes, Childhood obesity, Hip circumference, Overweight, Heel bone mineral density, Illnesses of father: diabetes, Snoring	28892062, 26551672, 25673412, 23563607, Neale B	*FTO*
rs11176018 ǂ	T	G	0.0176	0.004387	0.0350	0.01176	Birth weight	Neale B	*RNA5SP362*
rs11205802	T	C	-0.0202	0.004159	0.0024	0.01131	Mean corpuscular hemoglobin concentration, Red cell distribution width, Snoring	27863252, 27863252	*MACF1*
rs1136070 ǂ	T	C	0.0366	0.007184	-0.0634	0.02036	Snoring, Impedance of whole body, Platelet count	Neale B, 27863252	*HN1L*
rs11634019	T	C	0.0273	0.00438	0.0017	0.01170	Snoring	Neale B	*ISL2*
rs1182116 ǂ	T	C	-0.0147	0.003985	-0.0092	0.01063	NA	NA	*TYR*
rs12603115	T	C	-0.0181	0.003926	-0.0093	0.01053	Snoring	Neale B	*SKAP1*
rs12683343	G	A	0.0145	0.003942	-0.0135	0.01054	Body mass index, Weight, Hip circumference, Ever smoked, Daytime dozing or sleeping	Neale B, 28892062	*RP11-65N13.8*
rs12805133	G	A	0.0189	0.003964	0.0063	0.01398	Height, Impedance of leg left/right	Neale B	*SPTBN2*
rs13021737	G	A	-0.0218	0.005121	-0.0157	0.01058	Body Mass Index, Weight, Obesity class 1, Overweight, Diastolic blood pressure	28892062, Neale B, 23563607, 23563607	*TMEM18*
rs1403848	C	A	0.0157	0.003962	-0.0094	0.01062	Snoring	Neale B	*ROBO2*
rs1436047	G	A	-0.0254	0.00394	0.0055	0.01347	NA	NA	*RP11-354I13.1*
rs1444789	T	C	-0.0370	0.005042	0.0070	0.01052	Eosinophil count, Allergic disease asthma hay fever or eczema, Snoring, Wheeze or whistling in the chest in last year	27863252, 29083406, Neale B	*RP11-428L9.2*
rs1554654	T	C	-0.0181	0.003898	0.0110	0.01347	Body mass index, Weight	Neale B	*RP4-555D20.4*
rs17794954	T	C	-0.0225	0.005277	-0.0162	0.01053	Weight, Body mass index, Body fat percentage, Body mass index	Neale B 25673413	*LINC01524*
rs227731	T	G	-0.0260	0.003896	0.0109	0.01721	Nonsyndromic cleft lip, Orofacial clefts, Snoring	22863734, 20023658, Neale B	*C17orf67*

rs2277339	T	G	0.0451	0.006445	0.0003	0.01102	Mean corpuscular volume, Platelet crit, Height, Menopause age at onset, Basal metabolic rate, Whole body fat-free mass, Snoring	27863252, 28146470, 22267201, Neale B	*PRIM1*
rs2735309	T	C	-0.0209	0.004116	0.0017	0.01170	Snoring, Age at menarche, (No blood clot, bronchitis, emphysema, asthma, rhinitis, eczema or allergy diagnosed by doctor)	Neale B	*HMGN1*
rs2958153	G	A	-0.0315	0.004429	0.0094	0.01790	Snoring, Platelet count, Heel bone mineral density	27863252, Neale B	*PTGES3*
rs35445111	G	A	-0.0475	0.006817	0.0209	0.02951	Snoring	Neale B	*RNA5SP471*
rs4987719	T	C	0.0611	0.01068	-0.0082	0.01297	Snoring	Neale B	*BCL2*
rs543874	G	A	-0.0267	0.004938	-0.0163	0.01058	Body mass index, Weight, Obesity class 2	29273807, Neale B, 23563607	*SEC16B*
rs592333	G	A	0.0400	0.004068	0.0317	0.01247	Snoring, Impedance of arms	Neale B	*DLEU7*
rs6038517	G	A	0.0170	0.0046	0.0149	0.01085	NA	NA	*CASC20*
rs6113592	G	A	-0.0199	0.003998	0.0218	0.01343	Androgenetic alopecia early age of onset, Hair or balding pattern: pattern 2:4	Neale B	*CFTRP3*
rs6265	T	C	-0.0220	0.00501	-0.0089	0.01236	Body mass index, Overweight, Smoking behavior, Basal metabolic rate, Whole body fat mass, Snoring	28892062, 23563607, 20418890, Neale B	*BDNF*
rs6567160	T	C	0.0241	0.004574	0.0040	0.01166	Basal metabolic rate, Weight, Body mass index, Height,Suffer from nerves, Type II diabetes	28892062, 25673413, Neale B, 24509480	*RNU4-17P*
rs7005777	T	G	0.0175	0.00433	-0.0502	0.01050	Basal metabolic rate, Snoring, Whole body fat-free mass	Neale B	*AC105242.1*
rs7107532 ǂ	G	A	-0.0273	0.003886	0.0017	0.01208	Snoring, Comparative body size at age 10,	Neale B	*METTL15*
rs7138383	G	A	0.0171	0.004429	-0.0069	0.01536	Weight, Body mass index	Neale B	*C12orf42*
rs72904209	T	C	-0.0360	0.005858	0.0102	0.01076	Snoring, Forced expiratory volume, Forced vital capacity	Neale B	*LINC01876*
rs7715167	T	C	0.0239	0.004209	0.0218	0.01060	Height, Basal metabolic rate, Whole body fat-free mass	25282103, Neale B	*HMP19*
rs79932406	T	G	0.0161	0.003924	0.0016	0.01053	NA	NA	*XKR9*
rs806292	G	A	-0.0224	0.003905	0.1598	0.02174	White blood cell count	27863252	*DLEU1*
rs8176749ǂ	T	C	-0.0321	0.007433	0.0000	0.01079	Tumor biomarkers, Red blood cell count, Coagulation factor levels, Urinary metabolites H NMR features, Snoring, Malaria	23300138, 27863252, 23267103,	*ABO*

rs879620	T	C	-0.0156	0.003993	-0.0265	0.01506	Weight, Basal metabolic rate, Body mass index, Height	24586186, 23717212 Neale B, 28892062, 25282103	*ADCY9*
rs9526702	G	A	0.0239	0.005716	0.0233	0.01091	NA	NA	*RNASEH2B-AS1*
rs9783497	G	A	-0.0421	0.004022	-0.0107	0.01154	Height, Snoring	Neale B	*MSRB3*

Supplementary Table 3: all 39 single nucleotide polymorphisms (SNPs) to reach genome-wide significance in Campos et al. sleep apnoea (SA) meta-analysis, A1 is the effect allele, A2 is the non-effect allele, Beta and SE the effect size and standard errors for each SNP taken from the 23andMe replication of the Campos et al. meta-analysis Beta G and SE G are the respective effect sizes and standard errors for glaucoma taken from Craig, Han et al.. The sample size of 23andMe is 1477352, the effective sample size of the 23andMe SA cohort is 170817. Linked traits shows the main traits associated with the SNP, taken from Cambridge University's PhenoScanner. Source, PMID show the PubMed ID of the discovery genome-wide association studies that identifying the SNP-trait association; Neale B refers to the work of Ben Neale's lab, who ran and made publicly available genome-wide associations studies for all UK biobank traits (which can be downloaded here http://www.nealelab.is/uk-biobank/ [7]). The final column shows the closest gene to the given SNP. SNPs that were removed by HEIDI-outlier are marked with ǂ.

Table 4. Mendelian randomisation estimates of the association between sleep apnoea and glaucoma risk.

Method	beta	se	OR	OR 95% CI L	OR 95% CI U	Pval
GSMR	-0.050	0.057	0.952	0.850	1.065	0.388
IVW	-0.053	0.064	0.948	0.837	1.074	0.402
MR Egger	-0.191	0.188	0.826	0.572	1.193	0.315
Weighted median	-0.077	0.084	0.926	0.786	1.092	0.360
Simple mode	-0.004	0.177	0.996	0.705	1.408	0.984
Weighted mode	-0.119	0.143	0.887	0.670	1.176	0.411

*Table 1: All methods used the same 34 SNP instrument in **Supplementary Table 3** . Beta is the effect size for glaucoma for a x2 multiplicative increase in risk of SA for all methods. Also reported are the standard error of the beta (se), the odds ratio (OR; exponential of beta to give an OR representative for glaucoma given a doubling in odds of SA), and the lower and upper bound of 95% confidence interval (95% CI L and CI U respectively). IVW is inverse variance weighted, SA is sleep apnoea and GSMR is Generalised Summary-data-based Mendelian randomisation (MR).*

Supplementary Plot S1 - plot showing the correlation between sleep Apnoea and snoring

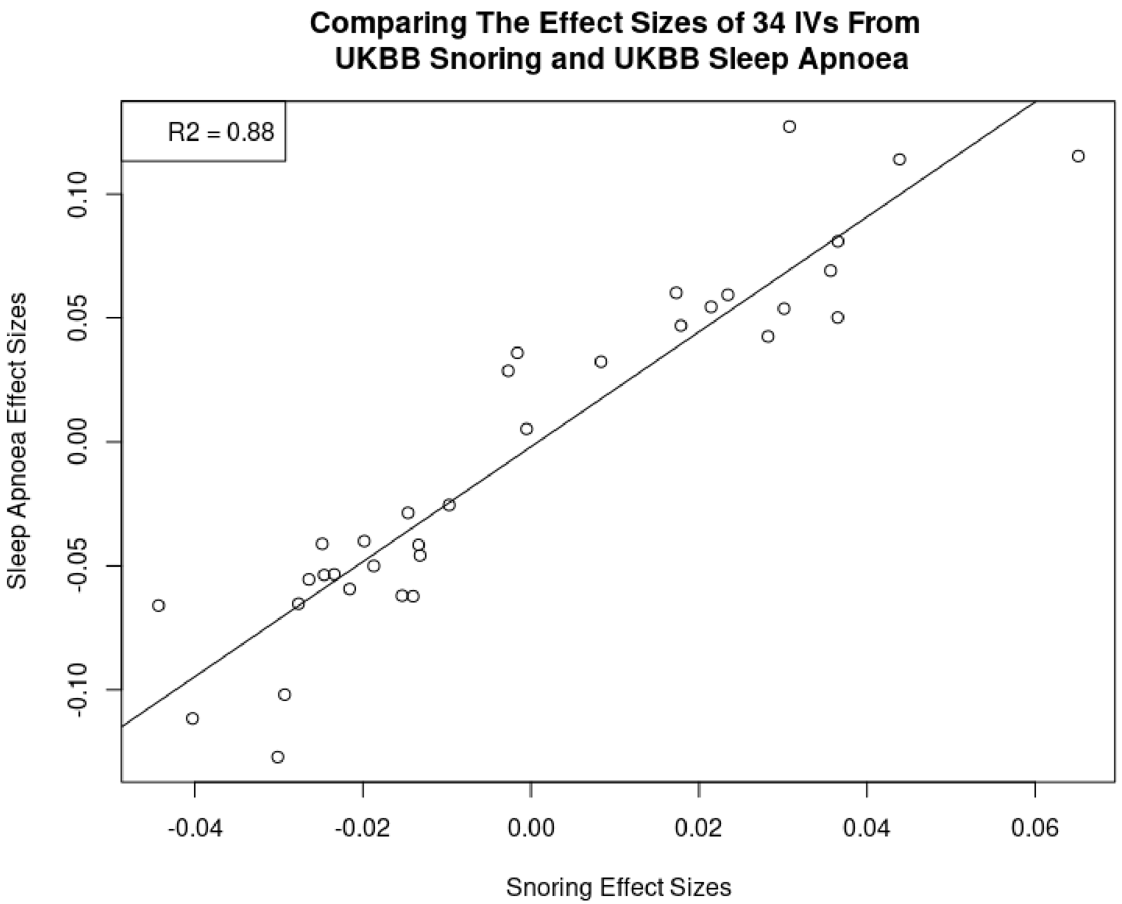

Supplementary plot 1 - shows the correlation between the effects of the 34 Ivs used in the MR with sleep apnoea and snoring

Supplementary References

1) Zhu, Z. et al. Causal associations between risk factors and common diseases inferred from GWAS summary data. Nat. Commun. 9, 224 (2018).
2) Hemani, G. et al. The MR-Base platform supports systematic causal inference across the human phenome. Elife 7, (2018).
3) Greenland, S. An introduction To instrumental variables for epidemiologists. Int. J. Epidemiol. 29, 1102 (2000).
4) Bowden, J., Davey Smith, G. & Burgess, S. Mendelian randomization with invalid instruments: effect estimation and bias detection through Egger regression. Int. J. Epidemiol. 44, 512–525 (2015).
5) Campos, A. I. et al. Genome-wide analyses in 1,987,836 participants identify 39 genetic loci associated with sleep apnoea. medRxiv (2020).
6) Craig, J. E. et al. Multitrait analysis of glaucoma identifies new risk loci and enables polygenic prediction of disease susceptibility and progression. Nat. Genet. 52, 160–166 (2020).
7) Neale, B. Neale Lab. http://www.nealelab.is/uk-biobank/ (2018).
